# Downy mildew symptoms on grapevines can be reduced by volatile organic compounds of resistant genotypes

**DOI:** 10.1038/s41598-018-19776-2

**Published:** 2018-01-26

**Authors:** Valentina Lazazzara, Christoph Bueschl, Alexandra Parich, Ilaria Pertot, Rainer Schuhmacher, Michele Perazzolli

**Affiliations:** 1 Department of Sustainable Ecosystems and Bioresources, Research and Innovation Centre, Fondazione Edmund Mach, Via E. Mach 1, 38010 San Michele all’Adige, Italy; 2 Center for Analytical Chemistry, Department of Agrobiotechnology (IFA-Tulln), University of Natural Resources and Life Sciences, Vienna (BOKU), Konrad-Lorenz-Straße 20, 3430 Tulln, Austria; 3 Centre Agriculture Food Environment, University of Trento, Via E. Mach 1, 38010 San Michele all’Adige, Italy

## Abstract

Volatile organic compounds (VOCs) play a crucial role in the communication of plants with other organisms and are possible mediators of plant defence against phytopathogens. Although the role of non-volatile secondary metabolites has been largely characterised in resistant genotypes, the contribution of VOCs to grapevine defence mechanisms against downy mildew (caused by *Plasmopara viticola*) has not yet been investigated. In this study, more than 50 VOCs from grapevine leaves were annotated/identified by headspace-solid-phase microextraction gas chromatography-mass spectrometry analysis. Following *P. viticola* inoculation, the abundance of most of these VOCs was higher in resistant (BC4, Kober 5BB, SO4 and Solaris) than in susceptible (Pinot noir) genotypes. The post-inoculation mechanism included the accumulation of 2-ethylfuran, 2-phenylethanol, β-caryophyllene, β-cyclocitral, β-selinene and trans-2-pentenal, which all demonstrated inhibitory activities against downy mildew infections in water suspensions. Moreover, the development of downy mildew symptoms was reduced on leaf disks of susceptible grapevines exposed to air treated with 2-ethylfuran, 2-phenylethanol, β-cyclocitral or trans-2-pentenal, indicating the efficacy of these VOCs against *P. viticola* in receiver plant tissues. Our data suggest that VOCs contribute to the defence mechanisms of resistant grapevines and that they may inhibit the development of downy mildew symptoms on both emitting and receiving tissues.

## Introduction

Plants are constantly exposed to environmental stressors and have evolved complex ways to defend themselves against pathogens, herbivorous arthropods, parasitic plants and neighbouring plant competitors^1^. Plants can produce a wide variety of volatile organic compounds (VOCs), which play a crucial role in the interaction of plants with other organisms and in the regulation of plant responses against biotic stresses^1,2^. VOCs constitute approximately 1% of plant secondary metabolites^3^ and are usually lipophilic molecules that can freely diffuse into the environment and pass biological membranes, thanks to their low molecular weight and high vapour pressure^4^. Based on their structure and biosynthetic pathways, plant VOCs can be divided into four main classes: terpenoids, phenylpropanoids/benzenoids, fatty acid derivatives and those derived from non-aromatic amino acids^2,3^. Volatile terpenoids are synthesized by the cytosolic mevalonic acid and plastidial methylerythritol phosphate pathway, which leads to the formation of carotenoids, mono-, di-, hemi- and sesquiterpenes^2^. Phenylpropanoid/benzenoid compounds are the second largest class of plant VOCs and they originate from phenylalanine through the shikimate/phenylalanine biosynthetic pathway^2^. Volatile fatty acid derivatives mainly derive from linoleic and linolenic acids through the lipoxygenase pathway^2^, while volatile amino acid derivatives contain nitrogen and sulphur and are synthesized from alanine, valine, leucine, isoleucine or methionine^3^.

The production and roles of plant VOCs in response to mechanical wounding or herbivore insects have been extensively investigated, but little is known about their involvement in defence mechanisms against pathogens^5,6^. Pathogen-induced VOCs typically consist of methyl salicylate (MeSA)^7–9^, mono- and sesquiterpenes^7,8,10–12^, heterocyclic compounds^7^, green leaf volatiles (GLVs) and ketones^10,11^. Three possible modes of action against pathogens have been attributed to plant VOCs, namely direct inhibition of microbial growth, induced and associational resistance^6^. For example, GLVs^13^ and β-caryophyllene^14^ directly inhibited bacterial growth and trans-2-hexenal reduced the germination of *Monilinia laxa*^15^ and *Botrytis cinerea* conidia^16^. Likewise, monoterpenes (limonene and β-linalool), nonanal and methyl jasmonate (MeJA) inhibited the germination of *Colletotrichum lindemuthianum*^6^, and esters (methyl propanoate and methyl prop-2-enoate) reduced the development of *Fusarium culmorum* and *Cochliobolus sativus*^17^. As a result of induced and associational resistance, VOCs can contribute to disease reduction in systemic parts of a locally attacked plants or in neighbouring plant receivers^6^. For example, VOC blends emitted by resistant plants^6,18^ induced defence-related processes in neighbouring plants, such as monoterpenes (α-pinene and β-pinene)^19^, MeSA^20^, MeJA and GLVs^21^. Finally, VOCs can be adsorbed to the cuticle of a receiver plant and these ‘sticky’ VOCs can persist on the leaf surface^22^, thereby exerting inhibitory effects against fungal pathogens and establishing the associational resistance^6^.

The involvement of plant VOCs in resistance mechanisms against pathogens is supported by specific VOC emission profiles in resistant and susceptible genotypes of maize to *Aspergillus flavus*^23^, citrus plants to ‘*Candidatus* Liberibacter asiaticus’^24^ and grapevine plants to *Plasmopara viticola*^12^. In the latter, the emission of the sesquiterpene and monoterpene classes was found to be more pronounced in downy mildew-resistant than in susceptible grapevine genotypes^12^, and the emission of a sesquiterpene [(E,E)-α-farnesene] was associated with the resistance induced by a sulphated laminarin against downy mildew^25^. Downy mildew, caused by the biotrophic oomycete *Plasmopara viticola*, is one of the most destructive diseases of the grapevine^26^. Resistance traits have been identified in wild grapevine species (*Vitis riparia*, *V. rupestris*, *V. amurensis* and *Muscadinia rotundifolia*) and the defence mechanisms against downy mildew have been investigated in resistant genotypes^27^. For example, physical (hairy and water repellent leaf surface) and chemical (phytoanticipins) barriers represent constitutive factors against pathogen infection^28^, while the accumulation of reactive oxygen species, pathogenesis-related proteins and non-volatile secondary metabolites (stilbenic phytoalexins and other antimicrobial phenolic compounds) has been shown to be a key post-inoculation mechanism involved in limiting *P. viticola* infection^29–31^. Although resistant genotypes produce some VOC classes after *P. viticola* inoculation^12^, identification of the underlying compounds and their functional role in grapevine resistance mechanisms have not yet been investigated. The aim of this study was to annotate/identify VOCs produced by resistant and susceptible grapevine genotypes in response to *P. viticola* inoculation using headspace-solid-phase microextraction gas chromatography-mass spectrometry analysis (HS-SPME/GC-MS) and to test their effects against downy mildew. Due to the obligate biotrophic lifestyle of *P. viticola*, inhibitory effects of VOCs can be tested only in the presence of host tissues and the final goal was to better understand the contribution of grapevine VOCs to limit downy mildew development in susceptible leaves.

## Results

### Profiles of VOCs detected in grapevine leaves

**Figure 1:**
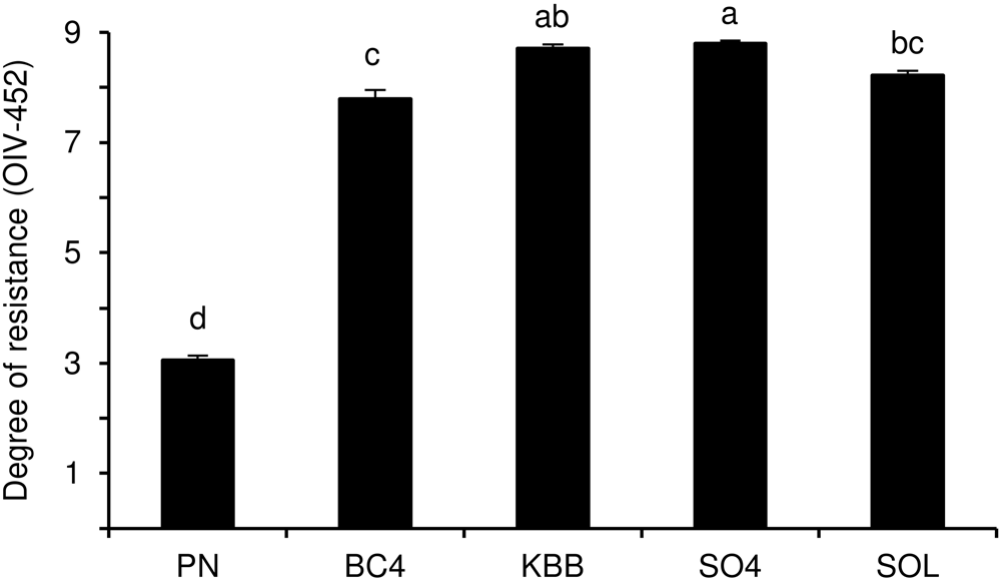
Degree of resistance of grapevine plants to downy mildew. Susceptible (Pinot noir; PN) and resistant grapevine plants [BC4, Kober 5BB (KBB), SO4, and Solaris (SOL)] were inoculated with *Plasmopara viticola* and the degree of resistance was assessed at seven days post inoculation according to the OIV-452 scores. Classes were assigned from the most susceptible (class 1) to the totally resistant (class 9) phenotype, according to the occurrence of sporangiophores and necrotic spots^61^. As Kruskal-Wallis test indicated no significant differences between two experiments (*p* > 0.05, n = 5 replicates per experiment), data from the two experiments were pooled. The pooled mean and standard error values of ten replicates (plants) are reported for each genotype. Different letters indicate significant differences among genotypes according to the Kruskal-Wallis test (*p* ≤ 0.05).

The evaluation of resistance levels confirmed a lower degree of resistance for the susceptible *V. vinifera* cultivar Pinot noir ENTAV 115 in both greenhouse experiments, as compared with the four downy mildew-resistant genotypes: BC4 [*M. rotundifolia* × *V. vinifera*^32^], Kober 5BB [*V. berlandieri* × *V. riparia*^33^], SO4 [*V. berlandieri* × *V. riparia*^34^] and Solaris [Merzling (Seyve-villard 5276 × Freiburg 379-52) × Geisenheim 6493 (Severnyi × Muscat Ottonel); http://www.vivc.de/] (Fig. 1). Specifically, leaves of susceptible Pinot noir plants showed dense sporulation of *P. viticola*, chlorotic spots and the absence of necrosis (mean OIV-452 score: 3), while those of BC4, Kober 5BB, SO4 and Solaris showed diffuse necrotic spots with sparse or absent sporangiophores (mean OIV-452 scores ranged from 7 to 9).

**Figure 2:**
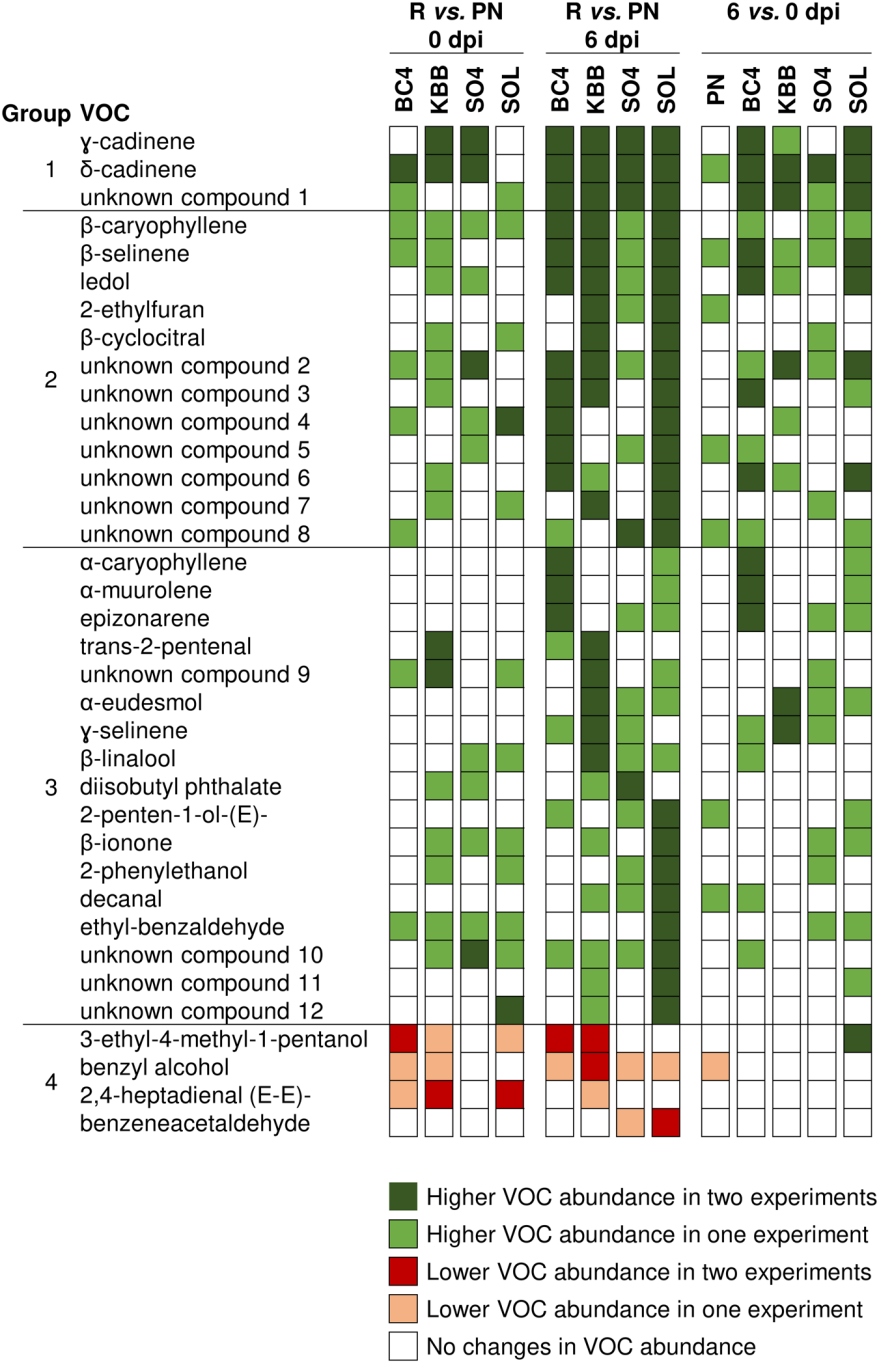
Profiles of volatile organic compounds (VOCs) of grapevine leaves. Susceptible (Pinot noir; PN) and resistant grapevine plants [BC4, Kober 5BB (KBB), SO4, and Solaris (SOL)] were inoculated with *Plasmopara viticola* and VOCs were detected before inoculation (0 dpi) and six days post inoculation (6 dpi) with *P. viticola* in two greenhouse experiments (Supplementary Tables S1 and S2). Three pairwise comparisons were carried out between VOC abundance in each resistant genotype and Pinot noir at 0 dpi (R *vs*. PN 0 dpi) or at 6 dpi (R *vs*. PN 6 dpi) and between 6 and 0 dpi for each genotype (6 *vs*. 0 dpi). Green and red cells indicate significantly higher and lower VOC abundance (Kruskal-Wallis test *p* ≤ 0.05 and fold change ≥ 1.5) in two (dark colour) or one (light colour) experiment, respectively. Metabolite groups were identified according to the VOC profiles: higher abundance in all resistant genotypes in both experiments at 6 dpi (Group 1), higher abundance in two or more resistant genotypes in both experiments at 6 dpi (Group 2), VOCs with a higher abundance in only one resistant genotype in both experiments at 6 dpi (Group 3), VOCs with a lower abundance in at least one resistant genotype in both experiments (Group 4) as compared with Pinot noir.

Leaf samples were collected immediately before inoculation (0 dpi) and six days post inoculation (6 dpi) with *P. viticola*, frozen in liquid nitrogen, ground to a fine powder and subjected to VOCs analysis according to the protocol optimized for grapevine leaves^35^ (Supplementary Fig. S1). A total of 56 and 52 VOCs were found in the five grapevine genotypes in the first and second experiment, respectively. In particular, 41 VOCs were annotated and 16 were found as unknown compounds according to the measured retention index (RI) of the HS-SPME/GC-MS analysis (Supplementary Tables S1, S2 and S3). Three pairwise comparisons were analysed to detect VOCs with significant changes in abundance (Kruskal-Wallis test *p* ≤ 0.05 and a fold change ≥ 1.5) between each resistant genotype and Pinot noir at 0 dpi (R *vs*. PN 0 dpi) and 6 dpi (R *vs*. PN 6 dpi) or between 6 and 0 dpi for each genotype (6 *vs*. 0 dpi). VOC profiles of the tested grapevine genotypes were mainly consistent in the two experiments, and they differed according to the grapevine genotypes and time points (Fig. 2, Supplementary Tables S1 and S2). Slight differences in VOC abundance occurred in resistant genotypes and the susceptible Pinot noir at 0 dpi (constitutive differences). On the other hand, the abundance of most of the annotated VOCs was consistently higher in resistant genotypes than in Pinot noir at 6 dpi in both experiments (post-inoculation differences). This is also reflected by the observation that most of the VOCs showed an increase in abundance at 6 dpi as compared with 0 dpi within each resistant genotype, but not in Pinot noir.

More specifically, VOCs were divided into six metabolite groups according to changes in abundance between resistant and susceptible genotypes consistently found in both experimental repetitions. The first metabolite group included two sesquiterpenes (γ-cadinene, δ-cadinene) and unknown compound 1, whose abundance was consistently higher in all resistant genotypes than in Pinot noir at 6 dpi in both experiments (Metabolite group 1). Moreover, γ-cadinene and δ-cadinene already showed higher constitutive levels in two (Kober 5BB and SO4) and three (BC4, Kober 5BB and SO4) resistant genotypes as compared with Pinot noir at 0 dpi in both experiments, respectively.

The metabolite group 2 summarises 12 compounds whose abundances were consistently higher in two or more resistant genotypes than in Pinot noir at 6 dpi in both experiments. β-caryophyllene, β-selinene and ledol and the two unknown compounds 2 and 3 were consistently more abundant in *P. viticola*-inoculated leaves of three resistant genotypes (BC4, Kober 5BB and Solaris) than in Pinot noir. The abundance of 2-ethylfuran and β-cyclocitral was higher in *P. viticola*-inoculated leaves of the resistant genotypes Kober 5BB and Solaris as compared with Pinot noir in both experiments. All the other unknown compounds (from 4 to 8) of this metabolite group showed higher levels in *P. viticola*-inoculated leaves of two of the four resistant genotypes as compared with Pinot noir at 6 dpi in both experiments.

The abundance of 17 VOCs was consistently higher in only one resistant genotype as compared with Pinot noir at 6 dpi in both experiments (Metabolite group 3). Specifically, α-caryophyllene, α-muurolene and epizonarene were consistently more abundant in *P. viticola*-inoculated leaves of BC4 than in Pinot noir. The abundance of trans-2-pentenal and unknown compound 9 was higher in Kober 5BB than in Pinot noir. Together with γ- and δ-cadinene, trans-2-pentenal and unknown compound 9 belonged to a group of four VOCs that were not only found to be induced by the pathogen inoculation but were also constitutively more abundant in Kober 5BB than in Pinot noir before inoculation. Moreover, α-eudesmol, γ-selinene and β-linalool showed higher abundance in Kober 5BB as compared with Pinot noir after *P. viticola* inoculation. *P. viticola* inoculation increased the abundance of a diester (diisobutyl phthalate) and eight VOCs [2-penten-1-ol-(E), β-ionone, 2-phenylethanol, decanal, ethyl-benzaldehyde and unknown compounds 10, 11 and 12] in SO4 and Solaris as compared with Pinot noir at 6 dpi, respectively.

In contrast to the high number of *P. viticola-*induced VOCs, two alcohols (3-ethyl-4-methyl-1-pentanol and benzyl alcohol) and two aldehydes [2,4-heptadienal (E-E)- and benzenacetaldehyde] were consistently less abundant in at least one resistant genotype and time point as compared with Pinot noir (Metabolite group 4). While the results described for metabolite groups 1–4 were consistent across both experiments, the profiles of 15 VOCs differed in the two experiments (Metabolite group 5). Moreover, five VOCs (α-copaene, germacrene B, germacrene D, dihydroactinidiolide and (+) -aromadendrene) and one VOC (octanoic acid) were detected only in the first or second experiment, respectively (Metabolite group 6).

### Effects of pure VOCs on downy mildew severity

**Figure 3:**
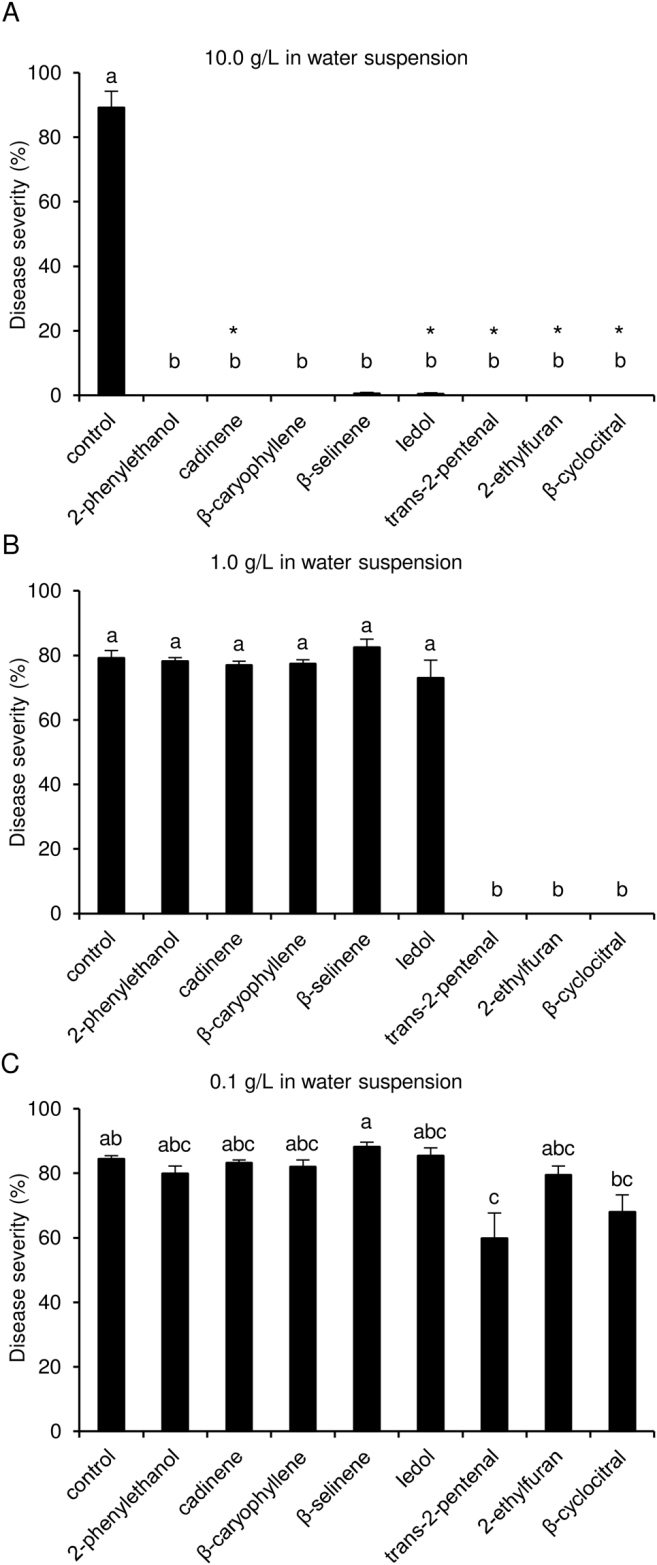
Effects of pure volatile organic compounds (VOCs) on downy mildew in water suspension. Leaf disks were inoculated with a *Plasmopara viticola* suspension without VOCs (control) or with 10.0 (**A**), 1.0 (**B**) and 0.1 (**C**) g/L of pure VOCs in water suspension (corresponding to 0.5, 0.05 and 5 × 10^−3^ mg/L in air volume, respectively). Five replicates (dishes with five disks each) were assessed for each treatment and the experiment was carried out twice. As the Kruskal-Wallis test indicated no significant differences between the two experiments (*p* > 0.05, n = 5 replicates per experiment), data from the two experiments were pooled. The pooled mean and standard error values of ten replicates are presented for each treatment. For each chart, different letters indicate significant differences among treatments according to the Kruskal-Wallis test (*p* ≤ 0.05). Asterisks indicate phytotoxic effects on leaf disks.

Eight VOCs were selected according to their consistent changes in abundance between resistant and susceptible genotypes in both experiments and they were tested as single pure compounds against *P. viticola* at different dosages in water suspension and air volume (Supplementary Fig. S1). More specifically, a mixture of ɣ- and δ-cadinene isomers was selected since they were consistently more abundant in all resistant genotypes at 6 dpi; three compounds (β-caryophyllene, β-selinene and ledol) and two compounds (2-ethylfuran and β-cyclocitral) were selected due to their consistently higher abundance in three (BC4, Kober 5BB and Solaris) and two resistant genotypes (Kober 5BB and Solaris) as compared with Pinot noir at 6 dpi respectively; trans-2-pentenal and 2-phenylethanol were selected as Kober 5BB (at 0 and 6 dpi) and Solaris (at 6 dpi) specific compounds, respectively. Leaf disks inoculated with the *P. viticola* sporangia suspension only (control) displayed severe sporulation at 6 dpi, while those inoculated with sporangia suspensions containing 10.0 g/L of each pure VOC had no disease symptoms (Fig. 3A). However, treatments with 10.0 g/L in water suspension of cadinene, ledol, trans-2-pentenal, 2-ethylfuran and β-cyclocitral caused phytotoxic effects on leaf tissues (diffuse chlorotic spots). Trans-2-pentenal, 2-ethylfuran and β-cyclocitral prevented downy mildew symptoms at the dosage of 1.0 g/L with no visible phytotoxic effects (Fig. 3B). At a VOC concentration of 0.1 g/L in water suspension, only trans-2-pentenal reduced downy mildew symptoms (Fig. 3C), with a disease reduction (efficacy) of 29.0 ± 9.2% (mean ± standard error, both expressed as a percentage), calculated according to the following formula: (disease severity of control disks – disease severity of VOC-treated disks)/(disease severity of control disks) × 100. No reduction in downy mildew severity was observed with pure VOCs at 0.01 g/L each (efficacy ranged from 0.0 ± 0.1% to 0.1 ± 0.0%) or with a blend of eight (2-phenylethanol, cadinene, β-caryophyllene, β-selinene, ledol, trans-2-pentenal, 2-ethylfuran, and β-cyclocitral) or three (trans-2-pentenal, 2-ethylfuran, and β-cyclocitral) pure VOCs at dosages of 0.1 (efficacy of 0.0 ± 0.0% and 0.3 ± 0.1%, respectively) or 0.01 g/L in water suspension for each compound (efficacy of 0.0 ± 0.0% and 0.2 ± 0.1%, respectively; Kruskal-Wallis test, *p* > 0.05).

**Figure 4:**
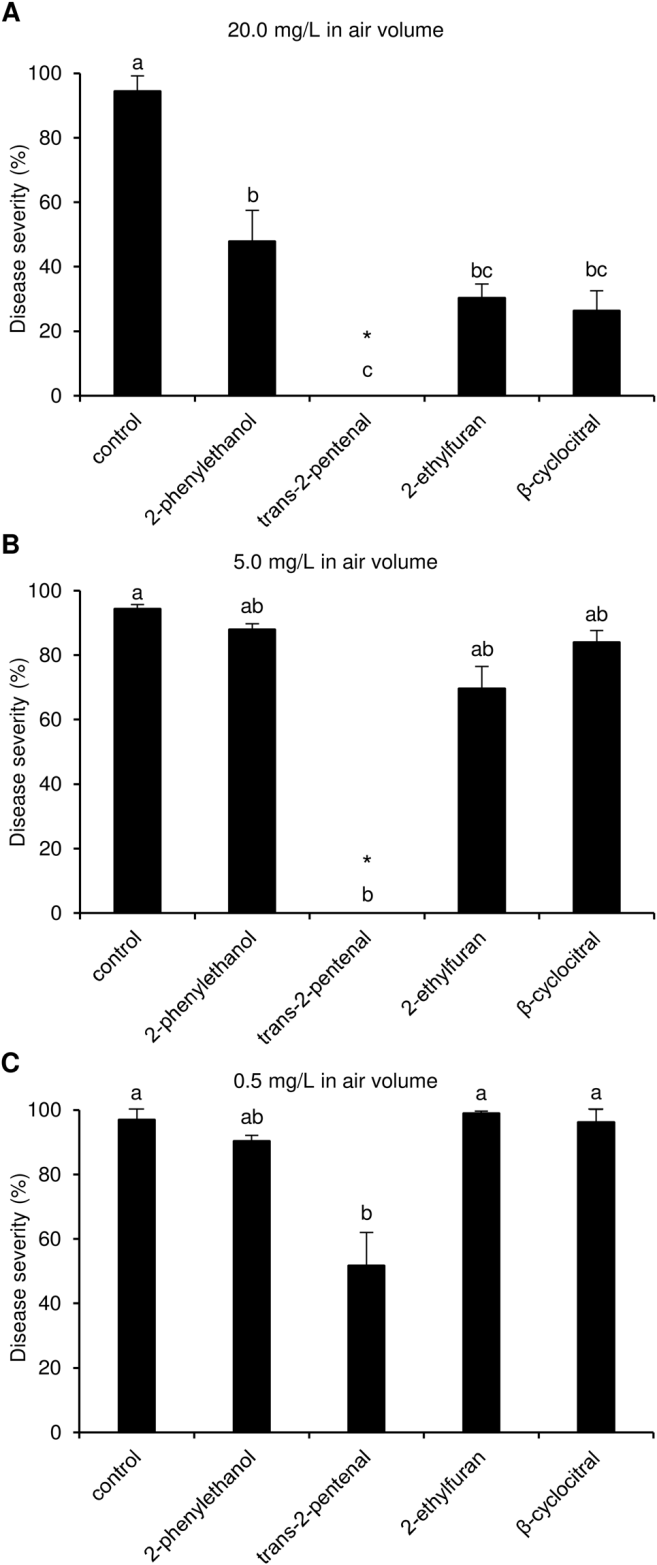
Effects of pure volatile organic compounds (VOCs) on downy mildew in air volume. Leaf disks were treated with water (control) or a pure VOC at concentrations of 20.0 mg/L (**A**), 5.0 (**B**) and 0.5 (black) mg/L in air volume, on a filter paper disk without contact with leaf tissues. Five replicates (dishes with five disks each) were assessed for each treatment and the experiment was carried out twice. As the Kruskal-Wallis test indicated no significant differences between the two experiments (*p* > 0.05, n = 5 replicates per experiment), data from the two experiments were pooled. The pooled mean and standard error values of ten replicates from the two experiments are presented for each treatment. For each chart, different letters indicate significant differences among treatments according to the Kruskal-Wallis test (*p* ≤ 0.05). Cadinene, β-caryophyllene, β-selinene and ledol (20.0 mg/L in air volume) did not affect downy mildew severity as compared with the control disks (Kruskal-Wallis test *p* > 0.05) and severity data are therefore not shown here. Asterisks indicate phytotoxic effects on leaf disks.

**Figure 5:**
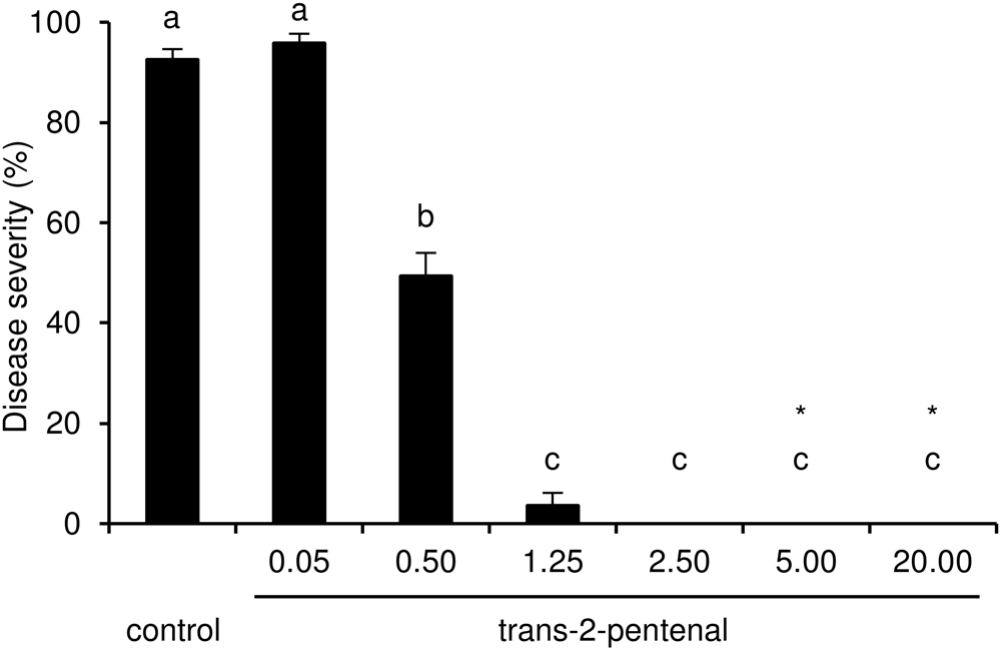
Effects of different concentrations of trans-2-pentenal on downy mildew in air volume. Leaf disks were treated with water (control) or trans-2-pentenal at different concentrations expressed in mg/L of air volume. Trans-2-pentenal was applied on a filter paper disk without contact with leaf tissues. Five replicates (dishes with five disks each) were assessed for each concentration and the experiment was carried out twice. As Kruskal-Wallis test indicated no significant differences between two experiments (*p* > 0.05, n = 5 replicates per experiment), data from the two experiments were pooled. The pooled mean and standard error values of ten replicates from the two experiments are presented for each treatment. Letters indicate significant differences among concentrations according to the Kruskal-Wallis test (*p* ≤ 0.05). Asterisks indicate phytotoxic effects on leaf disks.

The eight pure VOCs were also tested against *P. viticola* at different dosages in air volume without direct contact with the leaf tissue. These tests showed that 2-phenylethanol, trans-2-pentenal, 2-ethylfuran and β-cyclocitral reduced downy mildew symptoms at a dosage of 20 mg/L in air volume, while cadinene, β-caryophyllene, β-selinene and ledol did not (Fig. 4A). Leaf disks exposed to β-caryophyllene at a concentration of 50 mg/L in air volume showed phytotoxic effects, while those exposed to cadinene (78.0 ± 8.9%), β-selinene (85.6 ± 4.8%) and ledol (94.0 ± 0.9%) showed a disease severity comparable to control disks (98.8 ± 0.8%, Kruskal-Wallis test *p* > 0.05) and therefore these VOCs were not further used in activity tests. By lowering the concentration to 5.0 and 0.5 mg/L in air volume, only trans-2-pentenal reduced downy mildew symptoms with an efficacy of 100.0 ± 0.1% and 46.7 ± 10.3%, respectively (Fig. 4B and C). The dependence of efficacy on the concentration was tested in more detail for trans-2-pentenal, and at a concentration of 2.5 mg/L in air volume it was able to completely suppress downy mildew symptoms (efficacy 100.0 ± 0.1%) without any visible phytotoxic effects (Fig. 5).

**Figure 6:**
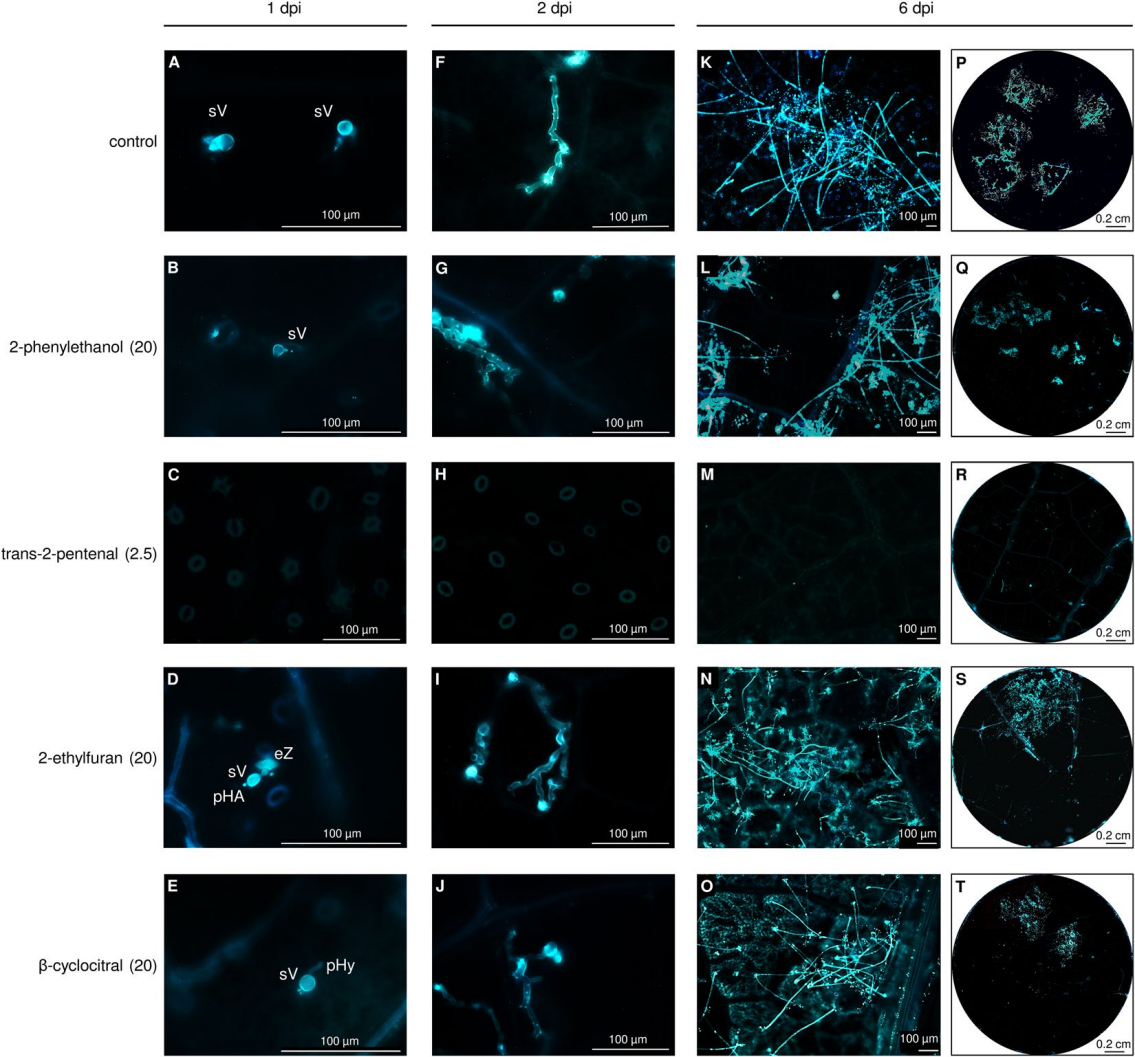
Effects of pure volatile organic compounds (VOCs) on downy mildew development. Leaf disks were treated with water (control), 2.5 mg/L (trans-2-pentenal) or 20 mg/L in air volume (2-phenylethanol, 2-ethylfuran or β-cyclocitral) on a filter paper disk without contact with leaf tissues. Disks were inoculated with *Plasmopara viticola* and the respective pure VOC was applied again to the filter paper disk. Pathogen development was monitored at one (**A–E**), two (**F–J**) and six (**K–T**) days post inoculation (dpi) using aniline blue staining. A representative leaf disk of ten is shown for each treatment and the experiment was carried out twice. Abbreviations: eZ, encysted zoospore; pHA, primary haustorium, pHy, primary hyphae; sV, substomatal vescicle. VOC concentrations, expressed as mg/L in air volume, are shown in brackets.

The four active VOCs in air volume (2-ethylfuran, 2-phenylethanol, β-cyclocitral or trans-2-pentenal) were further characterised with microscopic analysis, using the lowest concentrations at which the highest efficacy without visible phytotoxicity was observed (i.e. optimised concentrations), namely 2.5 mg/L in air volume of trans-2-pentenal and 20 mg/L in air volume of 2-ethylfuran, 2-phenylethanol or β-cyclocitral. Aniline blue-staining revealed marked differences between control and VOC-treated leaf disks after *P. viticola* inoculation (Fig. 6). At 1 dpi, the pathogen had already penetrated the stomata of control leaf disks, and encysted zoospores and substomatal vesicles were visible. The number of zoospores that had successfully entered stomata at 1 dpi was reduced in leaf disks treated with 2-phenylethanol, 2-ethylfuran or β-cyclocitral, while no infection structures were visible on trans-2-pentenal-treated disks. At 2 dpi, elongated and branched hyphae with haustoria were visible in control leaf disks, while primary haustoria and primary hyphae were occasionally visible in 2-phenylethanol-, 2-ethylfuran-, and β-cyclocitral-treated leaf disks. Again, no pathogen structures were visible in trans-2-pentenal-treated leaf disks at 2 dpi and sporulation was still not visible at 6 dpi. At 6 dpi, *P. viticola* mycelium had already spread to the parenchyma and produced sporangiophores in control leaf disks, while *P. viticola* sporulated areas were reduced in 2-phenylethanol-, 2-ethylfuran- and β-cyclocitral-treated samples.

### Effects of pure VOCs on *Plasmopara viticola* sporangia

**Figure 7:**
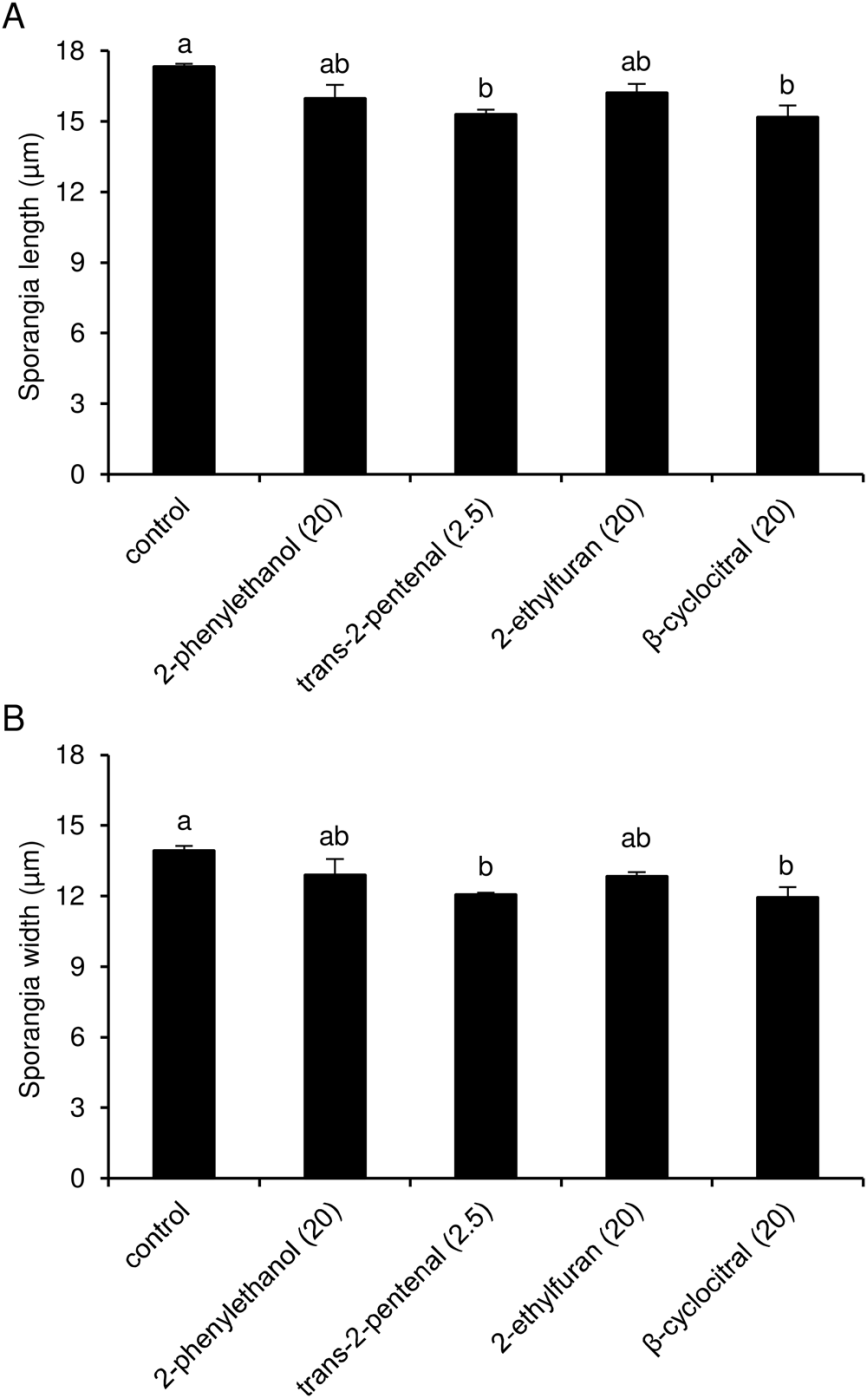
Effects of pure volatile organic compounds (VOCs) on *Plasmopara viticola* sporangia. Sporulated leaf disks were treated with water (control), 2.5 mg/L (trans-2-pentenal) or 20 mg/L in air volume (2-phenylethanol, 2-ethylfuran or β-cyclocitral) on a filter paper disk without contact with leaf tissues. Dishes were incubated overnight, after which *P. viticola* sporangia length (**A**) and width (**B**) were assessed. One hundred sporangia were measured for each replicate, five replicates (dishes) were assessed for each treatment and the experiment was carried out twice. As the Kruskal-Wallis test indicated no significant differences between the two experiments (*p* > 0.05), data from the two experiments were pooled. The pooled mean and standard error values of ten replicates are presented for each treatment. For each chart, different letters indicate significant differences according to the Kruskal-Wallis test (*p* ≤ 0.05). VOC concentrations, expressed as mg/L in air volume, are shown in brackets.

In order to assess the effects on *P. viticola* sporangia, the four active VOCs were tested at their respective optimised concentrations in air volume, as calculated from the experiments described above. Trans-2-pentenal and β-cyclocitral reduced sporangia length and width, while 2-phenylethanol and 2-ethylfuran did not (Fig. 7A and B). However, sporangia vitality was not affected by VOC treatments and the disease severity of disks inoculated with 2-phenylethanol- (77.8 ± 4.5%), 2-ethylfuran- (80.4 ± 2.7%), β-cyclocitral- (76.2 ± 10.4%) and trans-2-pentenal-treated sporangia (77.3 ± 11.4%) was comparable to that of control sporangia (79.8 ± 1.7%; Kruskal-Wallis test *p* > 0.05).

## Discussion

VOCs are known to play a crucial role in the communication between plants and other organisms^1,2^ and three possible modes of action against plant pathogens have been hypothesised so far^6^. More specifically, it has been shown that VOCs can directly inhibit pathogen growth, induce plant resistance mechanisms in neighbouring plants and mediate associational resistance by adsorption to the cuticle of receiver tissues^6^. The emission of some VOC classes has been demonstrated in resistant grapevines after *P. viticola* inoculation^12,36^, but annotation of their chemical structures and assessment of their functional roles in defence mechanisms have not yet been investigated. In agreement with previous literature on transcriptional regulation and accumulation of non-volatile metabolites^29,30^, VOC profiles were mainly related to post-inoculation mechanisms and significant increases in abundances were detected for 20 annotated VOCs at 6 dpi as compared with 0 dpi in all the four resistant grapevine genotypes tested (BC4, Kober 5BB, SO4 and Solaris). The role of VOCs in grapevine defence mechanisms was supported by the higher abundance in resistant genotypes as compared with the susceptible *V. vinifera* cultivar (Pinot noir) after *P. viticola* inoculation. For example, 11 terpenoids (α- and β-caryophyllene, α-muurolene, α-eudesmol, β-linalool, γ- and δ-cadinene, β- and γ-selinene, epizonarene and ledol) showed higher abundance in at least one resistant genotype as compared with Pinot noir, in agreement with the increased emission of this VOC class by *P. viticola*-inoculated plants previously shown by proton-transfer-reaction time-of-flight mass spectrometry analysis^12^. Moreover, the abundance of trans-2-pentenal, γ- and δ-cadinene also differed in resistant and susceptible genotypes before *P. viticola* inoculation, suggesting their involvement in constitutive defence mechanisms of resistant genotypes as well. Moreover, the majority of the unknown compounds (from 1 to 12) showed an increase in abundance in resistant genotypes as compared with Pinot noir at 6 dpi, but further studies are required to better identify the chemical structure and potential roles of these compounds.

Functional assays demonstrated that treatments with 2-ethylfuran, 2-phenylethanol, β-caryophyllene, β-cyclocitral, β-selinene and trans-2-pentenal in water suspensions reduced the development of downy mildew symptoms on Pinot noir leaf disks with no visible phytotoxic effects. Previous studies have indicated that VOCs are more abundant in the emitting leaf than in its surrounding gas space^6^ and they possibly accumulate in correspondence with the stomata^37^, suggesting that active VOCs can reach sufficiently high concentrations at stomatal infection sites to limit *P. viticola* infections. Thus, VOCs synthesized by resistant genotypes possibly contribute to resistance mechanisms by inhibiting downy mildew development in the plant emitter. Two active VOCs (β-caryophyllene and β-selinene) inhibited *P. viticola* only when applied in the water sporangia suspension, possibly due to the relatively low volatility of sesquiterpenes^38^, and they may form protective envelopes on the leaf surfaces of the plant emitter, with scarce migration to neighbouring plants. Moreover, four active VOCs (2-ethylfuran, 2-phenylethanol, β-cyclocitral and trans-2-pentenal) also prevented downy mildew symptoms on the susceptible Pinot noir leaf disks when applied in the air volume without direct contact with leaf tissues, indicating possible migration of these VOCs from plant emitters to neighbouring plants receivers. Previous studies have suggested that resistance induction can be impaired in detached leaves^39^ and this plant defence mechanism can show negligible effects on leaf disks^6^, indicating that these four active VOCs may contribute to associational resistance and reduce the severity of downy mildew symptoms on receiver plant tissues. Indeed, it has been reported that some VOCs can be adsorbed to the cuticle of the receiver and persist on its leaf surface^22^ and display inhibitory activities against phytopathogens through associational resistance mechanisms^6^. However, inhibitory effects of VOCs against downy mildew can be tested only in the presence of host tissues, due to the obligate biotrophic lifestyle of *P. viticola*. Thus, possible indirect effects of VOCs on host tissues cannot be totally excluded, such as the induction of plant resistance or slight phytotoxic effects. Indeed, sporangia vitality was not affected by the exposure to air treated with the four active VOCs, and only trans-2-pentenal and β-cyclocitral slightly reduced sporangia diameter. Thus, active VOCs could act against *P. viticola* once zoospores are released from sporangia and/or they possibly need the presence of host tissues to display the inhibitory activities. However, more precise functional and molecular studies on zoospore motility and grapevine resistance induction after VOC treatments are required to better understand the VOC activities against *P. viticola*. Moreover, stereochemical analyses are also needed to identify the stereoisomeric configuration of grapevine VOCs and the specificity of stereoisomers against downy mildew.

In the conditions applied in our study, trans-2-pentenal strongly inhibited downy mildew symptoms and was found in Kober 5BB plants before and after *P. viticola* inoculation. Trans-2-pentenal belongs to α,β-unsaturated aldehydes, which are categorised as GLVs^40^. Due to their chemical structure, α,β-unsaturated aldehydes can react with nucleophiles (such as protein sulphydryl or amino groups)^41^ and cause morphological deformations, collapse and deterioration of fungal structures^16^, as in the case of trans-2-hexenal against *B. cinerea*^16^ and *M. laxa*^15^. Therefore, the same mechanism of action can be hypothesised for trans-2-pentenal against *P. viticola* and can also explain the phytotoxic effects observed on grapevine leaf disks at dosages higher than 5 mg/L in air volume. Moreover, 2-phenylethanol, 2-ethylfuran and β-cyclocitral displayed moderate efficacy against downy mildew in air volume and they were mainly produced by one (Solaris) and two (Kober 5BB and Solaris) resistant genotypes after *P. viticola* inoculation, respectively. 2-phenylethanol (also known as benzeneethanol or phenylethyl alcohol) is present in plant essential oils^42^ and has previously been shown to have antimicrobial activity against *Escherichia coli* and *Rhizoctonia solanacearum*^42^, *Penicillium digitatum* and *P. italicum*^43^, *Candida albicans*, Gram-positive and negative bacteria^44^. Moreover, it has been reported that 2-ethylfuran accumulated during fatty acid oxidation in wild rocket^45^ and olive oil^46^, and it has nematicidal activity against *Meloidogyne incognita*^47^. Likewise, Ikawa, *et al*.^48^ and Ozaki, *et al*.^49^ respectively demonstrated that β-cyclocitral inhibited *Chlorella pyrenoidosa* and *Cyanobacterium microcystis*.

Sesquiterpenes (β-caryophyllene and β-selinene) reduced downy mildew symptoms in water suspensions and terpenes have already been classified as markers of genetic^12^ and induced resistance^25^ against grapevine downy mildew. Terpenes are generally recognised to contain antimicrobial metabolites^50^ and can interfere with mitochondrial membranes causing microbial cell death^51^. Infections of *Magnaporte oryzae* and *Pseudomonas syringae* pv. *maculicola* increased the emission of β-caryophyllene in rice leaves^52^ and tobacco plants^8^, respectively. Our results are also in agreement with Huang, *et al*.^14^, who demonstrated that β-caryophyllene inhibits *P. syringae* pv. *tomato* DC3000 in water suspensions and not in air volume (without direct contact). Likewise, β-selinene reduced downy mildew symptoms in water suspension and has previously been found in plant essential oils, with antimicrobial activities against *Staphylococcus aureus* and *C. albicans*^53^, *Bacillus licheniformis* and *Trypanosoma brucei brucei*^54^. Moreover, our results indicate that resistant genotypes can produce at least a further six terpenes (α-caryophyllene, α-eudesmol, α-muurolene, β-linalool, γ-selinene and epizonarene), one isoprenoid (β-ionone), one alcohol [2-penten-1-ol-(E)] and two aldehydes (decanal and ethyl-benzaldehyde), and some of these VOCs are known for their inhibitory activities against plant pathogens. For example, β-linalool inhibited *C. lindemuthianum*^6^ and *P. aeruginosa*^55^ and induced resistance against *Xanthomonas oryzae* in rice^56^. Likewise, β-ionone showed fungistatic activity against *C. musae*^57^ and decanal inhibited *Phytophthora infestans*^58^, indicating that several putative defence-related VOCs are synthesized by resistant genotypes in response to *P. viticola*.

In conclusion, *P. viticola* inoculation significantly increased the production of defence-related VOCs in resistant, but not in susceptible grapevine genotypes. Resistant grapevines accumulated six VOCs (2-ethylfuran, 2-phenylethanol, β-caryophyllene, β-cyclocitral, β-selinene and trans-2-pentenal) that reduced downy mildew symptoms on leaf disks and other putative defence-related VOCs (β-linalool, β-ionone and decanal) that possibly contribute to the inhibition of *P. viticola* infection. Moreover, downy mildew symptoms were impaired on leaf disks of susceptible grapevines exposed to air treated with 2-ethylfuran, 2-phenylethanol, β-cyclocitral or trans-2-pentenal, indicating that these four active VOCs possibly contribute to grapevine defence against downy mildew in systemic parts of a locally attacked plants or in neighbouring plants. Particularly, trans-2-pentenal was the most efficient VOC identified in this study and it represent a promising molecule from natural origin that could be further developed for downy mildew control of grapevine possibly with appropriate encapsulating formulations. Thus, VOCs could contribute to grapevine defence against downy mildew, but further metabolomic and transcriptomic analyses are required to investigate the possible VOC adsorption of the leaf cuticle and the possible indirect effects of VOCs on plant tissues, such as the activation of resistance mechanisms.

## Methods

### Inoculation of grapevine plants and assessment of disease severity

Grapevine rooted cuttings were grown under greenhouse conditions as described by Banani, *et al*.^59^. A *P. viticola* population was collected from an untreated vineyard in the Trentino region (northern Italy) and maintained by subsequent inoculations on *V. vinifera* Pinot Noir plants under greenhouse conditions^60^. Grapevines were inoculated with a suspension of *P. viticola* sporangia (2.5 × 10^5^ sporangia/mL) as described by Perazzolli, *et al*.^60^. The degree of downy mildew resistance was assessed at 7 dpi according to the OIV-452 descriptor^61^, and category scores from 1 (the most susceptible) to 9 (totally resistant) were assigned according to disease symptoms^62^. Ten replicates (plants) per genotype were assessed in a randomised complete block design. The experiment was carried out twice in two consecutive years (namely first and second experiment).

### Sample collection and VOC analysis

Leaf samples were collected immediately at 0 dpi and 6 dpi with *P. viticola*, to maximise the accumulation of non-volatile stilbenic phytoalexins^29,63^. Each sample comprised three leaves (from the fourth-sixth node) immediately frozen in liquid nitrogen, with five replicates (plants) being collected for each genotype and time point.

Samples were processed according to the protocol optimized by Weingart, *et al.*^35^ for grapevine leaves. Each frozen leaf sample was ground to a fine powder using a mixer-mill disruptor (MM301 Retsch) for 30 sec at 30 Hz, with pre-cooled 10 mL stainless steel beakers (Retsch) and a 9 mm stainless steel ball (Retsch). Leaf powder was transferred into 50 mL tubes and stored at −80 °C. Each sample (100 mg) was weighed in a 20 mL headspace vial (HS vials; Gerstel, Mülheim a.d. Ruhr), which was immediately sealed with a screw cap, assembled with a 1.3 mm silicone/PTFE septum (Supelco). As a quality control sample (QC sample), equal aliquots of each leaf sample were homogenised to determine technical variability^35^. Samples were measured in a randomised complete block design and a QC sample (100 mg) was analysed every eight grapevine samples.

VOCs were measured using HS-SPME/GC-MS analysis according to Weingart, *et al.*^35^. Briefly, each HS vial was placed in the auto-sampler at 15 °C (MPS2XL, Gerstel), after 20 min at 60 °C, a Divinylbenzene/Carboxen/Polydimethylsiloxane fibre (2 cm 50/30 µm; Supelco, Sigma-Aldrich) was inserted into the HS vial and the VOC extraction was carried out for 40 min at 60 °C. Analytes were desorbed in splitless mode at 250 °C for 2 min using an Agilent 6890 N gas chromatograph coupled to a quadrupole mass spectrometer 5975B Mass Selective Detector (MSD; Agilent Technologies). A non-polar DB-5MS column (Agilent Technologies) was operated at a constant 1 mL/min-flow of helium. The oven temperature was ramped from 35 °C to 260 °C with an increase of 5 °C per minute and the transfer line was set at 270 °C. Mixed alkane standard solutions for RI calibration were included in the sample list to ensure stable retention times and three SPME conditions were applied to obtain good peak shapes^35^.

Raw data were acquired with an Agilent MSD ChemStation (G1701EA E.02.00.493, Agilent Technologies) and the abundance of each VOC was calculated as the integrated peak area, expressed as counts per scan (cps), using MetaboliteDetector software (version 3.020151231 Ra-Linux)^64^. The mass spectrum deconvolution settings were: peak threshold of 4, minimum peak height of 4, deconvolution width (scans) of 5, required number of peaks set at 5. For compound annotation, deconvoluted mass spectra were compared with the NIST14 database (National Institute of Standards and Technology, http://www.nist.gov/) and with an in-house library of authentic reference standards. Compound annotation was achieved imposing a relative deviation of RI value lower than 2%^35^ and according to the highest mass spectrum similarity score, which was set to more than 70% after first successful annotation, in order to include low-abundance substances or substances where the deconvolution process did not lead to a complete elimination of interfering mass signals^35^. The in-house library was obtained with authentic reference standards in duplicate using the instrument and parameters reported above. VOCs with an average signal-to-noise ratio (S/N) lower than 10 (used as the limit of quantification^65^) were checked manually and only included in the data matrix if their abundance was significantly higher than 10 times S/N for at least one time point or genotype. To assess the technical precision of each experiment, the relative standard deviation of peak areas was calculated for every compound detected in the QC sample (RSD = 100*standard deviation/average of peak areas) and compounds with a RSD greater than 30% were discarded^66^. For each of the two experiments, five replicates (plants) were analysed per genotype and time point.

### Standard solutions and pure VOCs

Alkane standard solutions from C_8_ to C_20_ (40 mg/L each in hexane) and C_21_ to C_40_ (40 mg/L each in toluene) were purchased from Sigma-Aldrich. A standard solution from C_5_ to C_10_ was prepared using pure substances in a ratio resulting in narrow and symmetric peak shapes as described by Weingart, *et al*.^35^.

Pure VOCs were selected according to the SPME/GC-MS results, such as Benzenethanol, β-caryophyllene, trans-2-pentenal, 2-ethylfuran and β-cyclocitral (Sigma-Aldrich); cadinene (a mixture of ɣ-cadinene and δ-cadinene; (BOC Sciences); β-selinene and ledol (Xiamen Freede Industry). Pure VOCs were used in functional assays and for identity confirmation with HS-SPME/GC-MS analysis (Supplementary Fig. S2)

### Effects of pure VOCs against downy mildew

Leaves (from the fourth-sixth node) of Pinot noir plants were sterilised as described by Palmieri, *et al*.^67^. Leaf disks (18 mm diameter) were placed onto wet sterilised filter paper in Petri dishes, with the abaxial surface uppermost. Each pure VOC was diluted ten-fold in DMSO (Sigma-Aldrich) and serially diluted in distilled water to obtain the appropriate concentration for each treatment.

To assess the effects of pure VOCs against *P. viticola* in water suspension, each leaf disk was inoculated with five 5 μL-drops of a *P. viticola* suspension (2.5 × 10^5^ sporangia/mL), mixed with 0 (control), 0.01, 0.1, 1.0 and 10.0 g/L of the respective pure VOC (VOC-treated), calculated assuming the complete VOC dissolution in the water suspension. Dishes were incubated in the dark at 24 ± 1 °C overnight, then dried under a laminar hood and incubated for six days under greenhouse conditions as described by Palmieri, *et al*.^67^.

To assess the effects of pure VOCs on *P. viticola* in air volume, the respective pure VOC (0, 0.05, 0.5, 2.0 and 5.0 mg) was applied to a filter paper disk on the dish lid (without physical contact with the leaf tissue) as previously described^6,68^, corresponding to a concentration of 0 (control), 0.5, 5.0, 20 and 50 mg/L in air volume (VOC-treated) calculated assuming the complete VOC evaporation from the filter paper. Dishes were sealed with Parafilm (Beims) and incubated in the dark at 24 ± 1 °C for 24 h. Each leaf disk was inoculated with five 5 μL-drops of a *P. viticola* suspension (2.5 × 10^5^ sporangia/mL), the respective pure VOC was applied again to the filter paper disk in the appropriate concentration. Dishes were sealed with Parafilm and incubated in the dark at 24 ± 1 °C overnight. Leaf disks were dried under a laminar hood and incubated for six days under greenhouse conditions.

Disease severity was assessed at 6 dpi as a percentage of the leaf disk surface covered by sporulation^69^, calculated as the sum of the five inoculum drops. Each inoculum drop was scored as: 0%, no sporulation; 10%, scarce sporulation; 20%, dense sporulation. Five replicates (dishes) were assessed for each treatment and the experiments (i.e. in water suspension and air volume) were carried out twice.

Inoculated disks were collected at 1, 2 and 6 dpi and stained with aniline blue as reported by Lenzi, *et al*.^70^ by incubation in 1 M KOH at 95 °C for 15 min and staining with 0.05% aniline blue (Sigma-Aldrich) in 0.067 M K_2_HPO_4_ at pH 8 for 15 min Leaf disks (18 mm diameter) were observed under a LMD7000 microscope (Leica Microsystems) using an A4 filter (320–400 nm excitation, 400 nm dichroic mirror and 470 nm emission). Three leaf disks were analysed for each treatment and time point, and the experiment was carried out twice.

### Effects of VOCs on *Plasmopara viticola* sporangia

Sporulated leaves of Pinot noir plants were collected, leaf disks (18 mm diameter) were cut out and placed onto wet sterilised filter paper in Petri dishes, with the abaxial surface uppermost. The respective pure VOC was applied to a filter paper disk placed on the dish lid (without physical contact with the leaf tissue) at a concentration of 0 (control), 2.5 and 20 mg/L in air volume (VOC-treated), dishes were sealed with Parafilm and incubated at 24 ± 1 °C overnight. Sporangia were collected by washing five disks for each replicate in 2 mL of cold distilled water. Sporangia length and width were measured with a LMD7000 microscope (Leica Microsystems). One hundred sporangia were measured for each replicate (dish of five disks each), five replicates were assessed for each treatment and the experiment was carried out twice.

In order to assess sporangia vitality, each sporangia suspension (adjusted to 2.5 × 10^5^ sporangia/mL) was used to inoculate Pinot noir leaf disks as described above. Nine replicates (dishes with five disks each) were assessed for each treatment and the experiment was carried out twice.

### Statistical analysis

Each experiment was carried out twice and data on the degree of downy mildew resistance, disease severity and sporangia dimension were analysed using the Statistica 13.1 software (Dell). Each experimental repetition was analysed singularly and a Kruskal-Wallis test was used to demonstrate equivalent results in the two experiments (*p* > 0.05, non-significant differences between experimental repetitions). Data from the two experimental repetitions were pooled and a Kruskal-Wallis test was then used to detect significant differences among treatments (*p* ≤ 0.05).

VOC abundance was processed using an in-house R-script (R version 3.1.0). Data were inspected for outliers using the Dean-Dixon outlier test^71^. The Kruskal-Wallis test (*p* ≤ 0.05) and a fold change of VOC abundance ≥1.5 were set to classify VOCs with significant changes in abundance in three pairwise comparisons: (i) between each resistant genotype and Pinot noir before inoculation (R *vs*. PN 0 dpi) or (ii) six days post inoculation with *P. viticola* (R *vs*. PN 6 dpi) and (iii) between 6 and 0 dpi for each genotype (6 *vs*. 0 dpi).

## Electronic supplementary material

Supplementary Figures 1-2

Supplementary Table 1

Supplementary Table 2

Supplementary Table 3
