# Supplementary material for: Downy mildew symptoms on grapevines can be reduced by volatile organic compounds of resistant genotypes
Source: Sci Rep. 2018 Jan 26;8:1618. doi: 10.1038/s41598-018-19776-2 (PMC5786018; doi:10.1038/s41598-018-19776-2)
Supplement: Supplementary file 1 — Supplementary Figures 1-2 [file 41598_2018_19776_MOESM1_ESM.pdf]

Downy mildew symptoms on grapevines can be reduced by volatile organic compounds of resistant genotypes

Valentina Lazazzara[1,2], Christoph Bueschl[2], Alexandra Parich[2], Ilaria Pertot[1,3], Rainer Schuhmacher[2,+], Michele Perazzolli[1,+]

[1] Department of Sustainable Ecosystems and Bioresources, Research and Innovation Centre, Fondazione Edmund Mach, Via E. Mach 1, 38010 San Michele all'Adige, Italy. [2] Center for Analytical Chemistry, Department of Agrobiotechnology (IFA-Tulln), University of Natural Resources and Life Sciences, Vienna (BOKU), Konrad-Lorenz-Straße 20, 3430 Tulln, Austria. [3] Centre Agriculture Food Environment, University of Trento, Via E. Mach 1, 38010 San Michele all'Adige, Italy.

[+] These authors contributed equally to the coordination of this work.

Correspondence and requests for materials should be addressed to M.P. (e-mail: michele.perazzolli@fmach.it) or R.S. (e-mail: rainer.schuhmacher@boku.ac.at)

Supplementary Figures and Figure Legends

Figure S1. Overview of the experimental design. Leaf samples of the susceptible *Vitis vinifera* cultivar Pinot noir and four resistant *Vitis* spp. hybrids (BC4, Kober 5BB, SO4 and Solaris) were collected immediately before inoculation (0 dpi) and six days post inoculation (6 dpi) with *Plasmopara viticola*. Ground leaves were subjected to headspace-solid-phase microextraction gas chromatography-mass spectrometry analysis (HS-SPME/GC-MS) and two independent experimental repetitions were analysed to annotate/identify volatile organic compounds (VOCs). VOCs were selected according to their different levels in resistant and susceptible genotypes after pathogen inoculation and they were tested as single pure compounds in the functional assays. Two protocols were tested to asses the effect of pure VOCs against *P. viticola* i) in water suspension and ii) in air volume without direct contact with the leaf tissue.

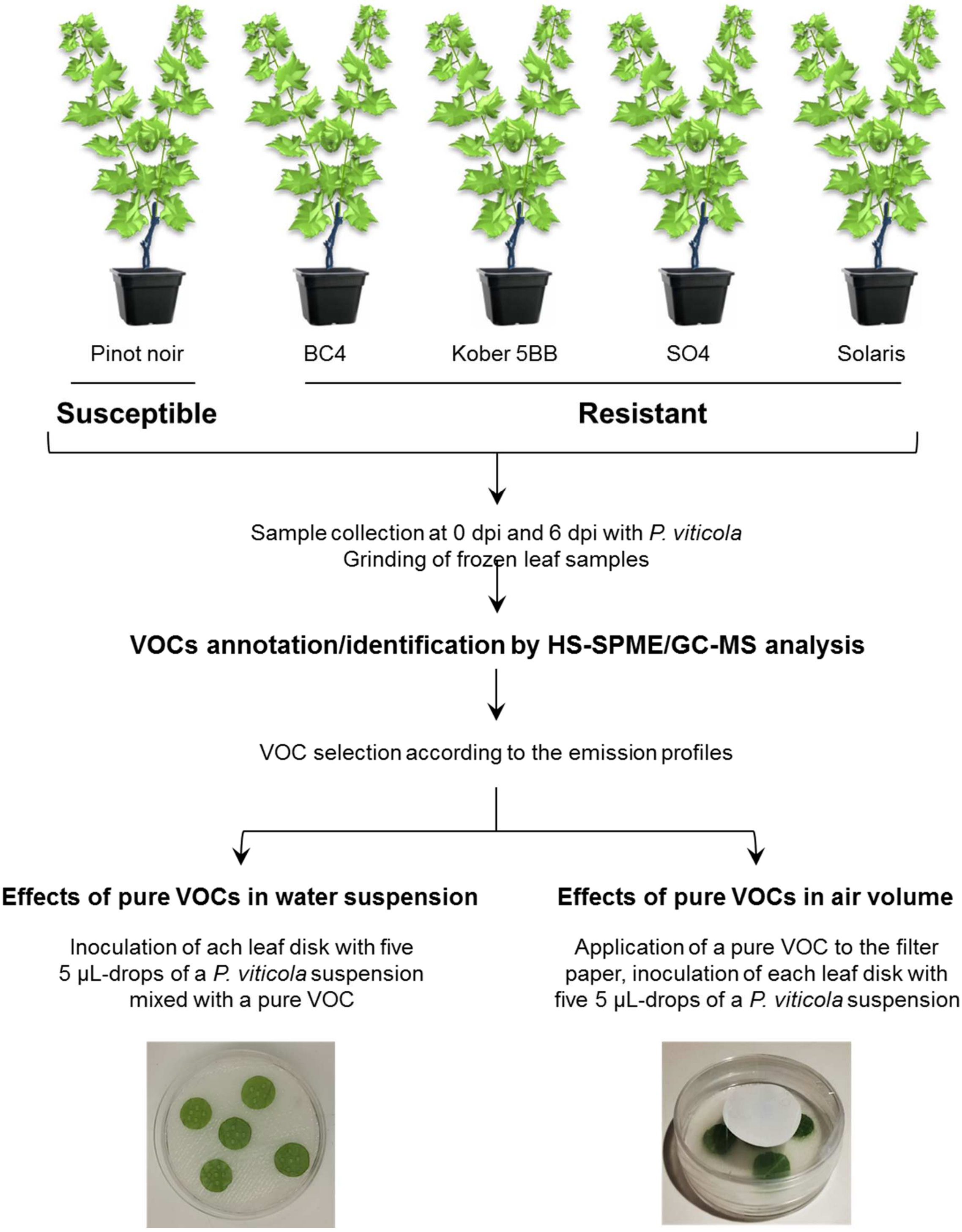

Figure S2. Comparison of the measured mass spectra of the volatile organic compounds (VOCs) in grapevine leaf samples with that of the corresponding pure VOC: 2-phenylethanol (A), ɣ-cadinene (B), δ-cadinene (C), β-caryophyllene (D), trans-2-pentenal (E), 2-ethylfuran (F), and β-cyclocitral (G). The mass spectrum similarity score and retention index values are reported for each VOC.

A   2-phenylethanol (similarity score 0.95)

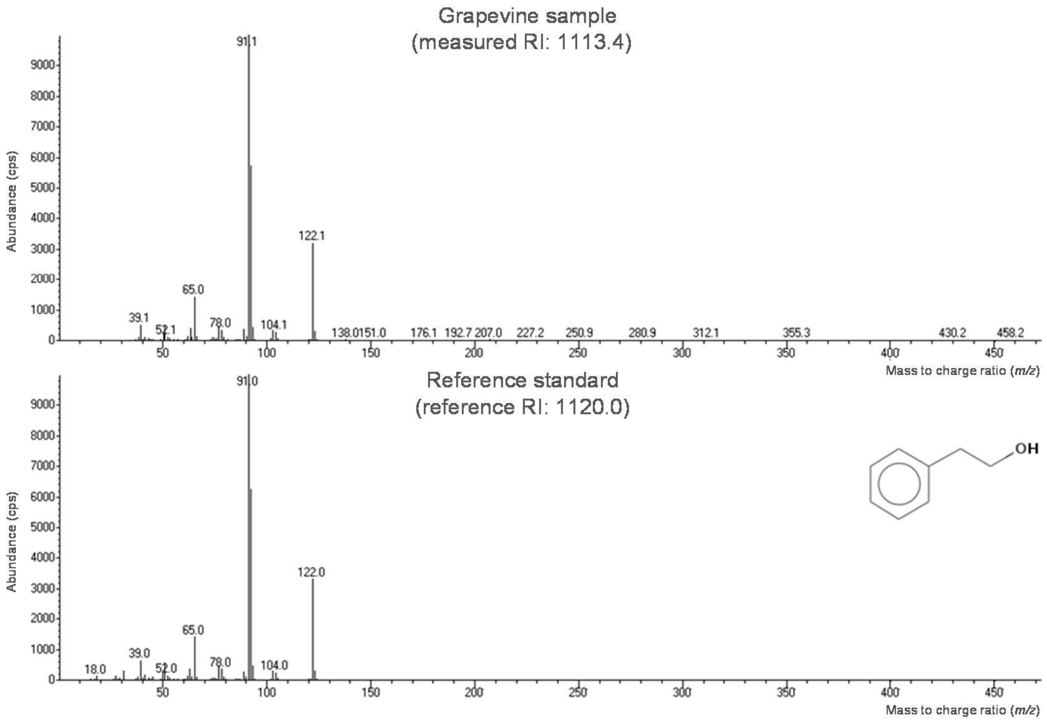

B   ɣ-cadinene (similarity score 0.93)

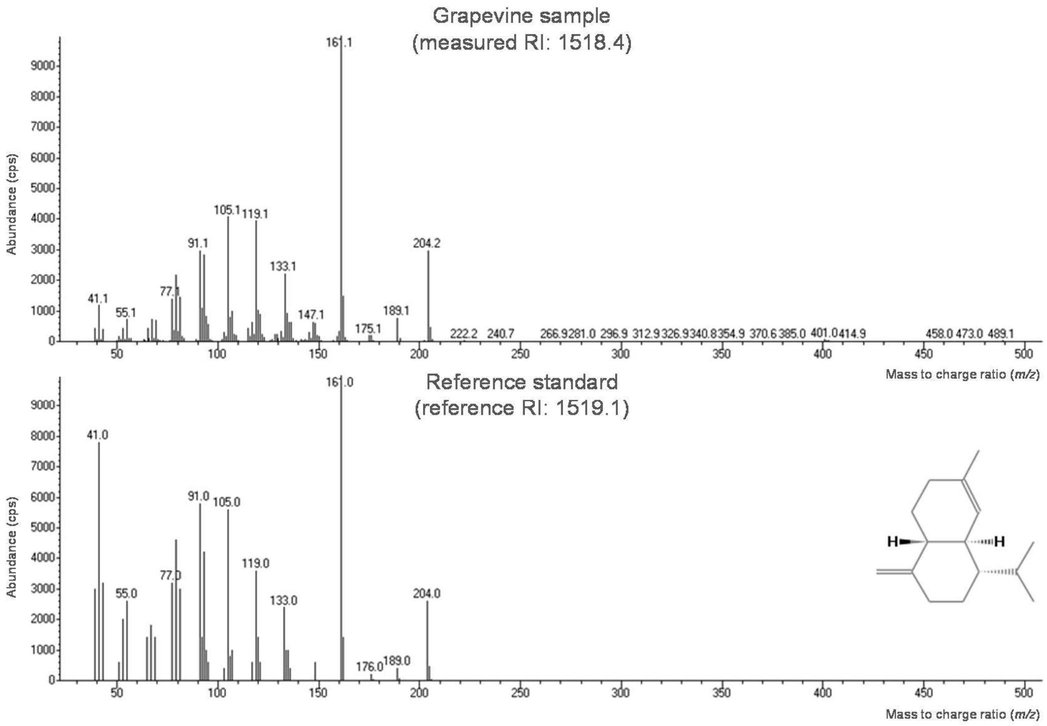

C δ-cadinene (similarity score 0.98)

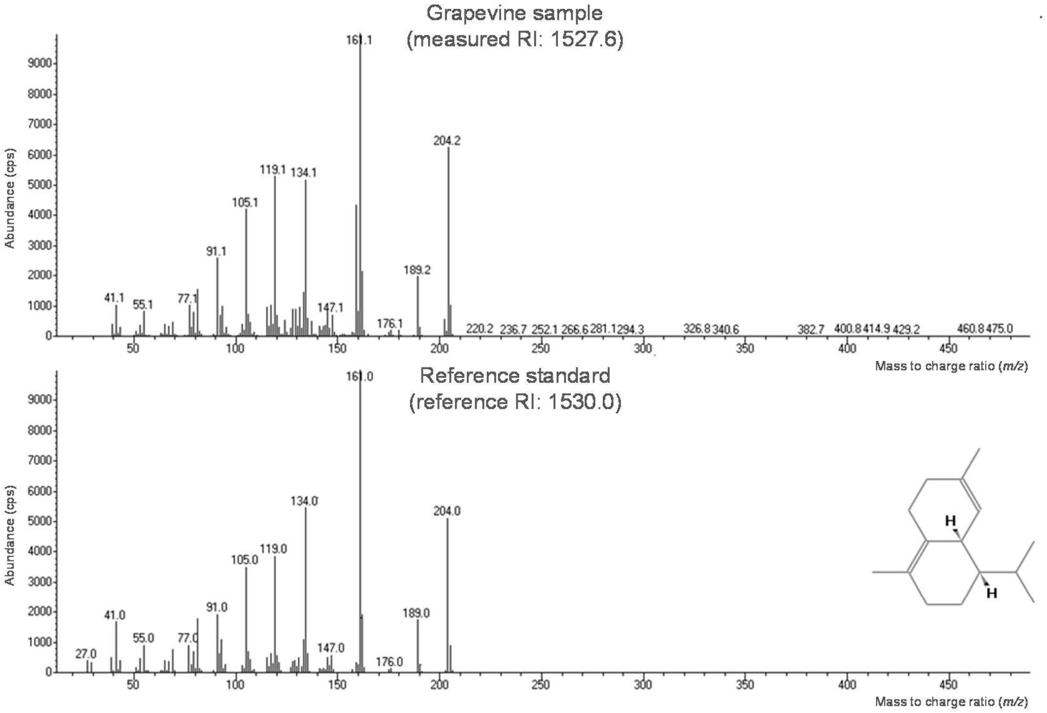

D β-caryophyllene (similarity score 0.94)

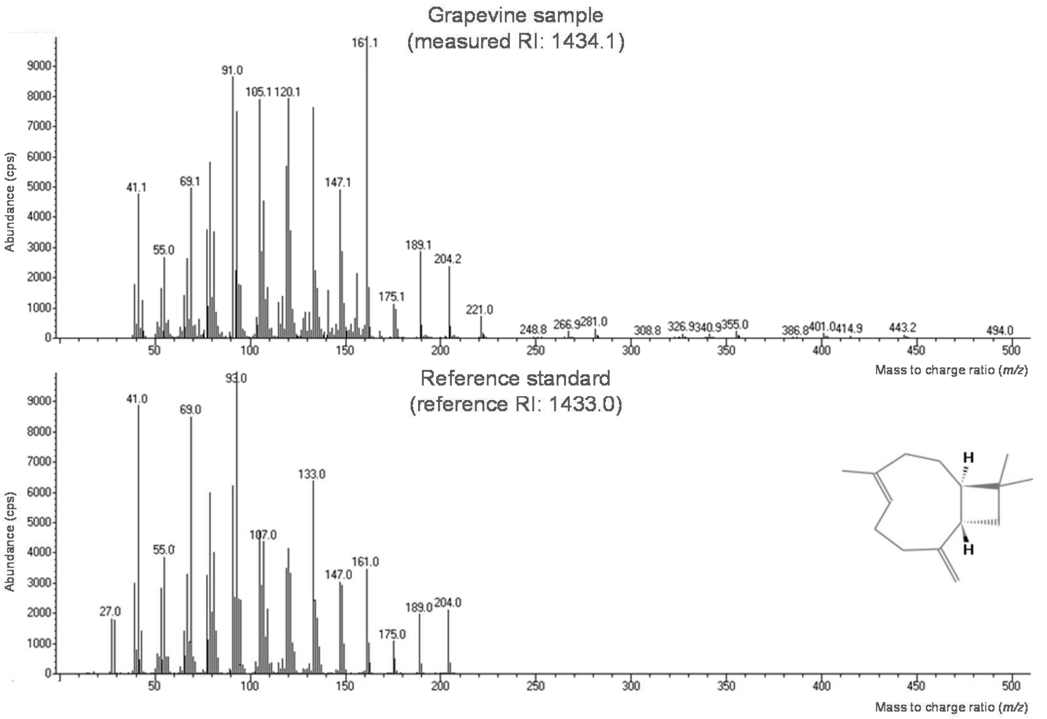

E

trans-2-pentenal (similarity score 0.81)

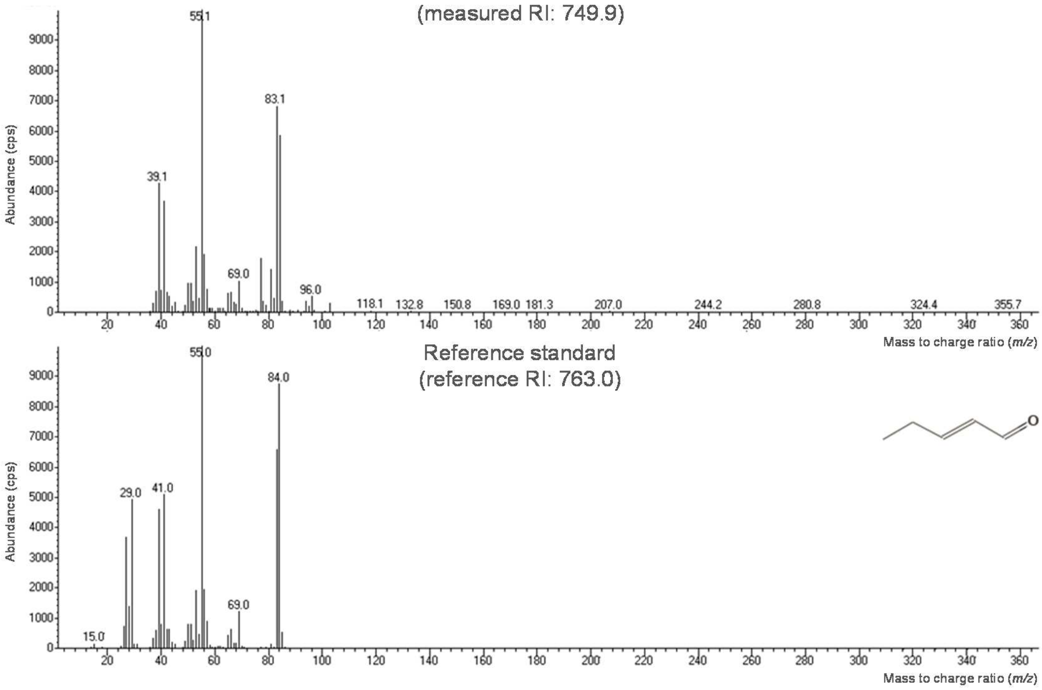

F

2-ethylfuran (similarity score 0.94)

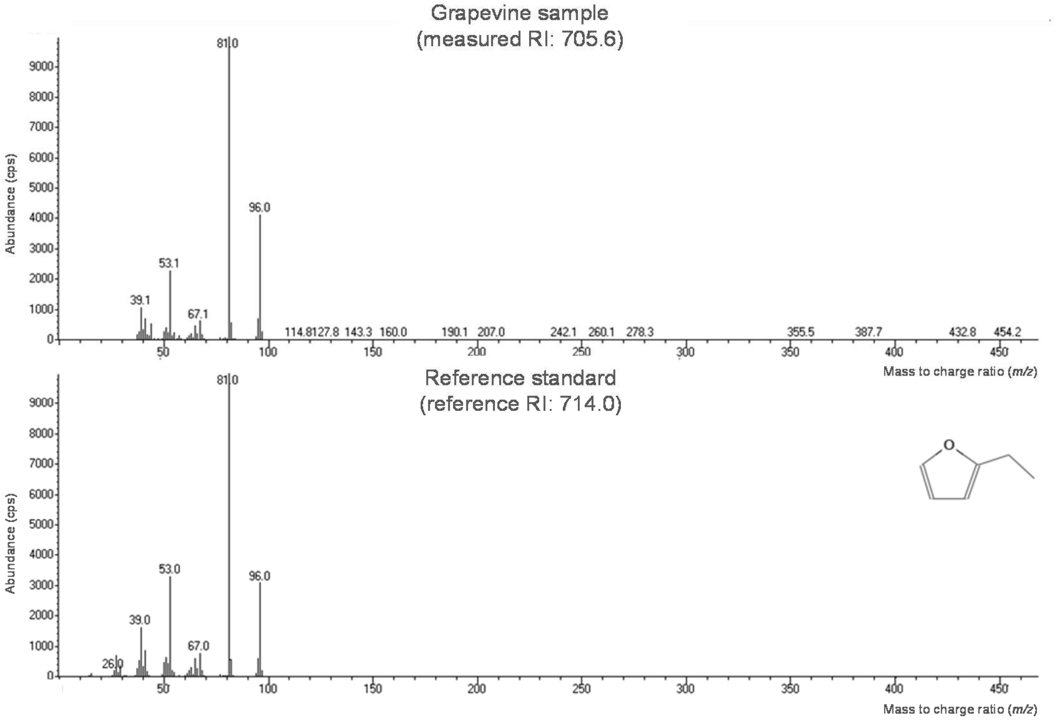

G β-cyclocitral (similarity score 0.94)

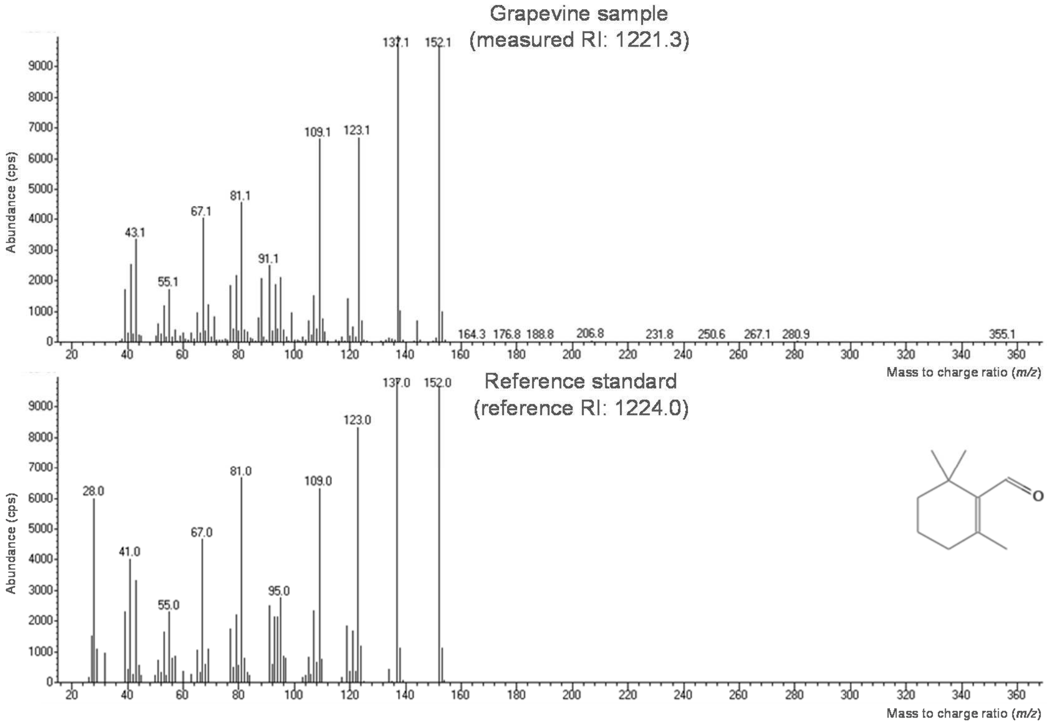

Supplementary Tables and Table Legends

Table S1. Volatile organic compounds (VOCs) detected by headspace-solid phase microextraction-gas chromatography-mass spectrometry from five grapevine genotypes in the first experiment.

Table S2. Volatile organic compounds (VOCs) detected by headspace-solid phase microextraction-gas chromatography-mass spectrometry from five grapevine genotypes in the in the second experiment.

Leaf samples were collected from susceptible [Pinot noir (PN)] and resistant [BC4, Kober 5BB (KBB), SO4, Solaris (SOL)] grapevine genotypes before inoculation (0 dpi) and six days post inoculation (6 dpi) with *Plasmopara viticola* and volatile organic compounds (VOCs) were measured using a headspace-solid phase microextraction-gas chromatography-mass spectrometry analysis (HS-SPME-GC-MS). Two independent repetitions of the experiment were carried out (namely first and second experiment).

Column A. VOCs were grouped in six metabolite groups according to their profiles in: VOCs with a higher abundance in all resistant genotypes as compared with Pinot noir in both experiments in at least one time point (Group 1); VOCs with a higher abundance in two or more resistant genotypes as compared with Pinot noir in both experiments in at least one time point (Group 2), VOCs with a higher abundance in only one resistant genotype as compared with Pinot noir in both experiments in at least one time point (Group 3); VOCs with a lower abundance in at least one resistant genotype as compared with Pinot noir in both experiments in at least one time point (Group 4); VOCs with different abundance profiles in the two experiments (Group 5); VOCs only found in the first or in the second experiment (Group 6).

Column B. Names of VOCs found in grapevine leaves using a HS-SPME-GC-MS analysis. Green cells represent VOCs with increased abundance consistent in the two experiments. Orange cells represent VOCs with decreased abundance consistent in the two experiments. White cells represent VOCs with increased or decreased abundance in one of the two experiments.

Column C. CAS Registry Numbers. Source: http://webbook.nist.gov/chemistry/
Column D. Measured retention index (Measured RI).
Column E. Retention index measured from an in-house library of authentic reference standards (Reference RI).
Column F. Measured retention time (Measured RT).

Columns G, M, W, AG, AQ. Mean of absolute peak area (abundance) expressed as counts per seconds (cps) of five biological replicates (plants) at 0 dpi.
Columns H, N, X, AH, AR. Standard error of absolute peak area (abundance) expressed as cps of five biological replicates at 0 dpi.

Columns I, O, Y, AI, AS. Mean of absolute peak area (abundance) expressed as cps of five biological replicates at 6 dpi.
Columns J, P, Z, AJ, AT. Standard error of absolute peak area (abundance) expressed as cps of five biological replicates at 6 dpi.

Columns K, Q, AA, AK, AU. Fold change (FC) values between 0 and 6 dpi for each genotype. Values are reported for significant changes ($p \leq 0.05$ of Kruskal-Wallis test and FC fold change > 1.5). Coloured cells represent consistent statistical differences in the two experiments (green and orange for VOC with increased or decreased peak area, respectively).

Columns L, R, AB, AL, AV. Asterisks indicated significant differences between 0 and 6 dpi for each genotype according to a Kruskal-Wallis test ($p \leq 0.05$) with a fold change of VOC abundances greater than 1.5. Coloured cells represent consistent statistical differences in the two experiments (green and orange for VOC with increased or decreased peak area, respectively).

Columns S, AC, AM, AW. Fold change (FC) values between of each resistant genotype against Pinot noir at 0 dpi. Values are reported for significant changes ($p \leq 0.05$ of Kruskal-Wallis test and FC fold change > 1.5). Coloured cells represent consistent statistical differences in the two experiments (green and orange for VOC with increased or decreased peak area, respectively).

Columns T, AD, AN, AX. Asterisks indicated significant differences of each resistant genotype against Pinot noir at 0 dpi according to a Kruskal-Wallis test ($p \leq 0.05$) with a fold change of VOC abundances greater than 1.5. Coloured cells (green and orange for VOC with increased or decreased peak area, respectively) represent consistent statistical differences in the two experiments.

Columns U, AE, AO, AY. Fold change (FC) values between of each resistant genotype against Pinot noir at 6 dpi. Values are reported for significant changes ($p \leq 0.05$ of Kruskal-Wallis test and FC fold change > 1.5). Coloured cells represent consistent statistical differences in the two experiments (green and orange for VOC with increased or decreased peak area, respectively).

Columns V, AF, AP, AZ. Asterisks indicated significant differences of each resistant genotype against Pinot noir at 6 dpi according to a Kruskal-Wallis test ($p \leq 0.05$) with a fold change of VOC abundances greater than 1.5. Coloured cells represent consistent statistical differences in the two experiments (green and orange for VOC with increased or decreased peak area, respectively).

Supplementary Table S3. Deconvoluted mass spectra of unknown compounds. Deconvoluted spectra were automatically generated using MetaboliteDetector software and the 20 most abundant ions are reported for each compound. Exceptions are unknown compounds 6 and 13, which exhibited less than 20 ions in their deconvoluted spectra. The intensity of the base peak (the most intense peak of the mass spectrum) was set to 100% and the intensities of the other most abundant ions of each deconvoluted spectrum are expressed relative to the base peak ion.
